# Immunopathogenesis in *Chlamydia trachomatis* Infected Women

**DOI:** 10.5402/2011/436936

**Published:** 2011-11-24

**Authors:** Maria Teresa Mascellino, Priscilla Boccia, Alessandra Oliva

**Affiliations:** Department of Infectious Diseases, Policlinico Umberto I, Viale del Policlinico 155, 00161 Rome, Italy

## Abstract

We examine the *Chlamydia trachomatis* (Ct) immunopathogenesis on the basis of the complex interaction between host immune response and virulence microorganism factors. Ct infection can be asymptomatic or may produce an inflammation elicited and preserved by reinfections or persistent infections. We discuss the host polymorphisms that, with their anti- or proinflammatory effects, determine the course of the disease. We also took into account the inflammation process following the *Chlamydia* illness and the role of both CD4 cells producing IFN-**γ** and CD8 cells with their cytokines production. The crucial role of Ct-hsp60 and the double activity (either damaging or preserving from some kinds of tumors) of anti-Ct-hsp60 antibodies are considered.

## 1. Introduction

*Chlamydia trachomatis *(Ct) is an obligate intracellular bacteria, and it results to be the most common sexually transmitted disease that, other than being asymptomatic and therefore unrecognized and untreated, promotes an acute or chronic inflammation causing tissue damage, pelvic inflammatory disease (PID), infertility, and ectopic pregnancy.

Latest international estimates show that around 92 million new cases of *Chlamydia *infection occur every year [1]. Most of *Chlamydia *goes undiagnosed, and its infection is often asymptomatic and can persist for long periods. Studies on the natural course of untreated *C. trachomatis* lower genital tract infections in women show spontaneous clearance rates of 30% in the first weeks to months, 50% in 1 year, 80% in 2 years, and 94% in 4 years [2]. Although this is often the case, chlamydial infection induces an intense and chronic inflammation.

The clearance of the microorganisms depends on both a normal immune response and an antibiotic treatment. However, some women are not able to clear the pathogen adequately and become asymptomatic. Repeated infections can be even more damaging for women, because they cause serious sequelae for the genital apparatus.

The principal findings concerning *Chlamydia trachomatis *immunopathogenesis include the following points:

**(1) The Normal Immune Response to Infections**

The host immune response to infection and how this relates to disease is still not completely understood. The immune response to Ct in infected women is involved in both immunity and pathology. A strong adaptive immune response is able to develop and increase the chlamydial virulence and to worsen the clinical course of the illness. This response is a key mechanism involved in controlling or eliminating the infection having a double-edged nature which can be both protective and tissue damaging.

**(2) The Host Susceptibility**

Host response greatly affects the outcome of Ct disease influencing and determining the pathology. The host response is not able to contrast or control the Ct infection. In fact, if the microorganism is not adequately treated, it remains for long periods in the infected subjects [2]. In other words, host response contributes to determine the microorganism pathology. Indeed, not only the characteristics of the pathogen, but also host genetics play an important role in determining susceptibility to infections and severity of disease. In recent years, the importance of host genetic polymorphisms in Ct-related pathologies has been demonstrated [3–6].

**(3) The Heat Shock Protein (hsp60)**

Ct-hsp60 has been shown to be an important target for the immune response linked to complications [7]. Antibodies to Ct-hsp60 have been suggested as markers of chronic inflammation and may, therefore, be good predictors for the risk of tubal pathology [8]. The findings of elevated human serological responses to Ct-hsp60 in individuals with severe diseases have been confirmed for genital tract illness as well as for trachoma [9]. The precise role of Ct-hsp60 (similar to human-hsp60) and anti-Ct-hsp60 antibody is still unclear.

Granted that chlamydial infection is based on the above points, our paper will address the immunopathology, the host susceptibility, and the crucial role of Ct-hsp60 in the pathogenesis.

***Chlamidia trachomatis *and Immunopathogenesis**

Chronic Ct infection is characterized by genital tract inflammation and mucosa infiltration of the full range of inflammatory cell types with a predominant amount of lymphocytes. However, and this is uncommon when considering chronic inflammation, there is a consistent neutrophilic component [5, 6]. The rate of inflammation and the consequent development of disease is known to be multifactorial: indeed, it involves the virulence of different strains of Ct, the environmental cofactors and the host immune response. Ct infection strongly induces and maintains both innate and acquired immune response. However, rarely the immune response to Ct results in clearance of the infection. This is due to the ability of Ct of evading the host's immune system [1, 3–6].

## 2. Innate Immune System

**Figure 1:**
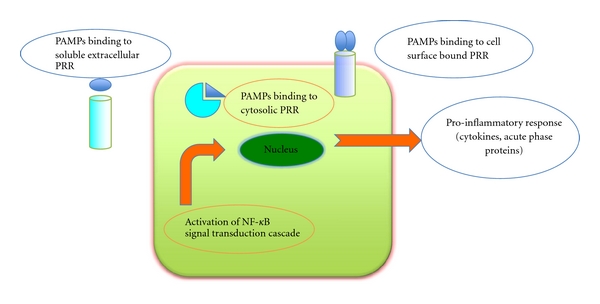
Modified from den Hartog et al. 2006 [3]. The innate immune response starts by binding of pathogen-associated molecular patterns (PAMPs) to the cells receptors (PRRs). This activates the nuclear factor (NF)-*κ*B that binds to specific DNA sequences in the nucleus, inducing the production of proinflammatory cytokines.

Innate immune system is a general, nonspecific system which is the first line of defense against pathogens. Ct infects the columnar epithelial cells of the endocervix of women. At the site of infection, an intense inflammation occurs attracting macrophages, neutrophils, dendritic cells (DCs), natural killer (NK) cells, T-lymphocytes, B cells, and so forth. After infection, epithelial cells produce various pro-inflammatory mediators including CCL5, CXCL16, CXCL 10, CXCL 1, IL-1*α*, IL-8, IL-12, IL-6, GM-CSF (granulocyte-macrophage colony stimulating factor), TNF (tumor necrosis factor), and GROa (growth-related oncogene) that induce and augment the cellular inflammatory response thus stimulating direct damage to the tissues and producing an increased expression of endothelial adhesion molecule that helps the attraction of immune cells [5]. Resident macrophages also contribute to early release of cytokines and chemokines. Both epithelial cells and circulating cells of the innate immune system possess receptors called pattern of recognitions (PRRs). The two most important families of PRRs are the Toll-like receptors (TLRs) and the nucleotide-binding oligomerization domain proteins (NODs). These receptors recognize and bind particular molecular antigens of the pathogen (PAMPs—pathogen-associated molecular patterns). PRRs of epithelial cells or of circulating cells are found in the extracellular, in the intracellular compartments (NOD), or bound to cell surface (TLR). Binding of NOD or TLR to PAMPs induces a series of reactions, which lead to the activation of the NF-KB (Nuclear factor) signal transduction cascade that binds to the nuclear DNA promoting and increasing the production of proinflammatory cytokines (Figure 1). Infected epithelial cells also release matrix metalloproteases (MMPs) that contribute to cell damage and scar formation.

Neutrophils, NK cells and monocytes are recruited in the infected tissue. MMPs and elastase are also produced by neutrophils thus contributing to pathology [5].

### 2.1. Toll-Like Receptors (TLRs) and (Nucleotide-Binding Oligomerization Domains) NODs

TLRs may be involved in the pathology of STIs (sexually transmitted infections). They act as pathogen-recognition receptors that enable cells to recognize chlamydial structural elements. In *Chlamydia *pathogenesis, TLR2 and TLR4 are mostly involved with TLR2 required for IL-8 secretion. Activation of these receptors was dependent on live, replicating bacteria, because it has been demonstrated [10] that the treatment of infected cells with antibiotics or UV eliminated the induction of IL-8 secretion suggesting that TLR2 is actively engaged in signalling from this intracellular location. A dominant role for TLR2 compared with TLR4 has been confirmed at least in the recognition and response to *Chlamydia muridarum *in the murine genital tract [11]. TLR2 is recruited to intracellular *Chlamydiae* and is required for cellular activation (determined by IL-8 measurement) during infection. In human cells, TLR2 is the PRR for the *C. trachomatis* component peptidoglycan, and it is mainly expressed in the tubes and cervix. On the contrary, TLR4 is the PRR for Ct components lipopolysaccharide (LPS) and heat shock protein, and it is mainly expressed in the tubes and endometrium and less or not at all in the endocervix [3, 6]. Clamydial heat shock protein 60 acts via TLR4 to activate NF-KB and increase IL-8 secretion. TLR1, TLR3, TLR5, and TLR6 are also present in the human female genital tract, but they do not recognize Ct-PAMPs. This suggests that the above TLRs may play a role in the host defense against non-Ct infections [12, 13].

NOD proteins are intracellular PPRs. They include two subclasses (NOD1 and NOD2) and are able to recognize intracytoplasmatic bacterial PAMPS such as LPS and peptidoglycans. Because Ct is an intracellular pathogen containing LPS and peptidoglycan, the role of intracellular NOD in recognition of *C. trachomatis *is crucial [14]. Binding of an NOD to its PAMP also activates the NF-KB signal transduction cascade, which initiates the immune response.

It is hypothesized that the risk of late sequelae increases with the number of genetic variations in NODS or TLRs [3]. Carrying multiple genetic variations in PRRs plays a role in the development of *C. trachomatis*-associated tubal pathology. den Hartog et al. [3] showed a higher risk in carriers of at least two genetic variations (73%) as compared with carriers of less than two variations (33%). Therefore, carrying multiple genetic variations rather than a single one is determinant for the risk of late sequelae. In this context, the nucleotide polymorphisms in PRRs (TLRs and NODs) in women with chlamydial infection suggest that pattern recognition receptors are involved in the progression of the infection [6]. The presence of TLRs and NODs in the genital tract is reported in Table 1.

### 2.2. NAP (Natural Antimicrobial Peptides and Defensins)

The immune innate system produces other key mediators such as the NAPs (natural antimicrobials peptides) and the defensins both playing an important role in the pathogen elimination. NAPs, which include secretory leukocyte protease inhibitors (SLPI) and elafin, are released at epithelial surfaces and disrupt the membranes of many microbial pathogens [15].

SLPI and elafin are able to contrast the action of MMPs (metalloproteases), thus contributing to the prevention of the damage due to an over production of proteases by epithelial cells and neutrophils. SLPI inhibits the neutrophil elastase, the trypsin, and other damaging products; elafin is mainly directed against neutrophil elastase and proteinase 3 [16]. SLPI and elafin are expressed in the female genital tract. The effect of NAPs and especially SLPI are contrasted by anti-SLPI antibody in the endometrial epithelial cells. These data suggest that SLPI is included in the endometrial and Fallopian tube epithelium innate immune response [17, 18]. SLPI and elafin show a variety of anti-inflammatory effects even in other tissues probably due to their antiproteinase or antibacterial effects.

Leucocytes and epithelial cells produce a variety of human defensins (human *β*-defensins HBD and *α* defensins-HD5) that result to be present in the endometrial epithelium [19]. Being present at key sites, they have been reported to be involved in the innate immune response during pregnancy in order to maintain sterile the uterus environment [20]. Innate immune system competence is of critical importance in preventing microbial penetration [6]. In fact, in women's genital tract, we can distinguish the sterile upper tract (endometrium and Fallopian tube) and the nonsterile lower tract (vagina and cervix). They have a compartmentalized innate immune response: in vagina and endocervix, although they are colonized by a variety of commensal bacteria, infections are relatively uncommon suggesting effective containment or efficient elimination of pathogens. Infection of the endometrium and tube occurs when the microorganism breaches the cervical barrier and ascends to the upper genital tract. Knowing in advance the innate immunity in the genital tract is decisive, because it will inform us on the interventive strategies to protect women against disease and eventually to treat the infection [21].

## 3. Acquired Immune System

The acquired (or adaptative) immune system is a specific system that develops after the first contact with a pathogen. Macrophages and both dendritic cells (plasmacytoid DCs and myeloid DCs) are able to express on their surface bacterial antigens bound to major histocompatibility complex and to serve as antigen presenting cells (APC), which is critical for the activation of the adaptative immune system. Plasmacytoid dendritic cells (pDCs) were reported to be mainly recruited in women with inflammation in the genital tract or in those having fertility disorders [1]. The response to APC is stronger than innate immune response of epithelial or circulating cells, inducing a more marked inflammatory response. A Ct infection evokes a vigorous local and systemic acquired humoral and cell-mediated response. 

### 3.1. Humoral Immunity

In the humoral arm, B-lymphocytes are activated by APC and develop into plasmacells which are able to produce antibodies such as Immunoglobulins (Igs).

The dominant immunoglobulin isotype found in the cervicovaginal fluid of the female genital tract is IgG rather than secretory IgA. These antibodies can neutralize the antigen or directly destroy the pathogen inactivating extracellular elementary bodies (EBs) [5]. It has been shown [1] that Ct-specific antibodies do not generally correlate with resolution of infection in individuals, but they are correlated with severe sequelae such as tubal infertility, ectopic pregnant, and PID. Moreover B-lymphocytes can serve as APCs for T-lymphocytes. As a consequence, although antibodies can help in clearance of infection, their major role is in the enhancement of Th1 activation [3].

In female, the prevalence of IgG and IgA antibodies towards Ct-MOMP antigen (major outer membrane protein) is mainly found in subjects with primary chlamydial infections, whereas the presence of antibodies against Ct-hsp60 and Ct-hsp10 is significantly higher in patients with recurrent or persistent infections. The dominant Ct-hsp60 and Ct-hsp10 antibodies are found in all the situations, where major fertility disorders are reported [21–24].

### 3.2. Cell-Mediated Immunity

In the cell-mediated arm, T-lymphocytes are activated by APCs (cells of innate immune system or B lymphocytes). Most T-lymphocytes are T-helper cells (Th). The Th cells can be subdivided in Th1 and Th2 subclasses both producing proinflammatory cytokines. The mononuclear cells produce IL-12 and induce the differentiation of naïve T-cell into T-helper1 (Th1). Th1 cells secrete IL-12, IFN-*γ*, and IL-2 which support the cell-mediated system increasing the level of inflammatory processes, whereas Th2 subclass produces IL-4, IL-5, IL-6, and IL-10 which support the humoral system. The relative contributions of the two classes of Th-cells determine whether the cell-mediated or the humoral immunity is predominant.

Th1-type responses (producing IFN-*γ*) play a role in the resolution of infection, whereas Th2-type responses involved in the humoral immunity is crucial for scarring [4]. In fact, patients with severe scarring are found to show high levels of antichlamydial antibody. Holland et al. [25] reported that patients with more severe diseases often produced Th2-type patterns of cytokines. Murine studies also support the hypothesis that Th1 cells are involved in protection, while Th2 cells are involved in persistence and disease [26]. NK cell production of interferon (IFN-*γ*) drives CD4T cells towards the Th1-IFN-*γ* producing phenotype then leading to protection of infection. T-cell production of IFN-*γ* may also inhibit intracellular chlamydial replication possibly inducing a persistent infection. In fact, IFN-*γ* has been shown to delay the cycle of *Chlamydia* so that the reticulate bodies persist longer and could result in persistent, unapparent infection and then contribute to immunopathogenesis [1].

In many studies [5, 27], it has been reported that the resolution of genital chlamydial infection is dependent on the influx of IFN-*γ* producing CD4+ Th1 cells. These cells, that are essential for the host defense, may also cause collateral tissue damage and scarring [28]. Production of IFN-*γ* by peripheral blood mononuclear cells (PBMCs) stimulated with Ct-hsp60 strongly correlates with protection against ongoing Ct infections although there is no information regarding the development of late sequelae. Defining the specific responses that promote tissue damage and differentiating them from those that lead to benign resolution of infection is an important ongoing research goal.

In the presence of chronic or repeated infections, CD4 and CD8 cells infiltrate in larger numbers than neutrophils, B cells and plasmacells do, and this recurrent inflammatory response is eventually responsible for the ultimate pathological effects in Ct infections.

The acute inflammatory response occurs, at the same extent, in both initial and repeated infections, whereas T-cells are predominant in the latter ones. In the repeated infections, a lymphoid follicle containing all kind of cells (B-cells, macrophages, neutrophils, and T cells) is formed in the deep stroma. The more rapid and intense response observed in the reinfections depends on the massive calls of chronic inflammatory leucocytes, but, in this context, the recruited infiltrate contains *Chlamydia*-specific immune cells that amplifies the response promoting further tissue scarring [4–6, 8–29].

**Figure 2:**
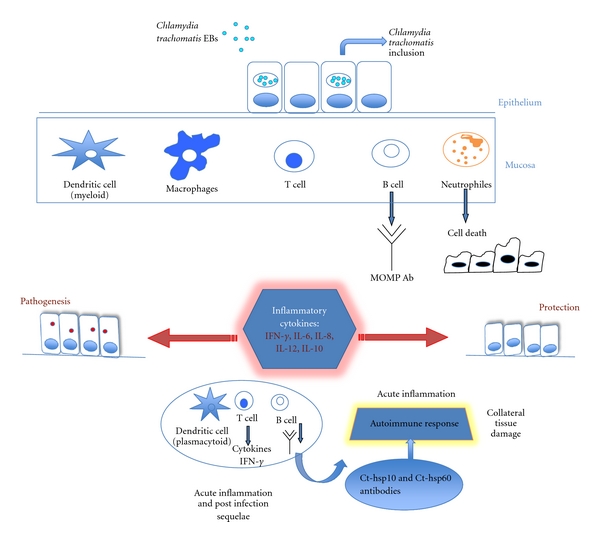
Modified from Agrawal et al. 2009 [1] (see text for details).

The immune response in human females during *C. trachomatis *infection of the cervical epithelial cells is reported in Figure 2.

### 3.3. Regulatory T-Cells (Tregs)

Regulatory T-cells (Tregs), the action of which is to downregulate immunity, have been extensively studied in the pathogenesis of allergy, autoimmunity, and inflammatory diseases; recently, many researchers have been focused on their role during Ct infection [30]. Natural Treg cells, expressing CD4+CD25+ and the transcription factor FOXP3 (forkhead box P3), are selected in the thymus and move to the periphery. In the periphery, CD4+ T cells can be induced to become Tregs (called adaptative Treg cells), and hence secrete IL-10 or TGF-*β* (transforming growth factor *β*) or both. High levels of IL-2 induce the proliferation of Tregs and also contribute to their efficiency and fitness. Furthermore, it is known that bacterial heat-shock protein 60 (hsp60) and flagellin enhance the suppressive functions of CD4+CD25+ T-cells through the production of IL-10 and TGF-*β*. Treg cells inhibit the function of both Th1 and Th2 CD4+ T cells and their production of cytokines; additionally, their role is to suppress the CD8+ T cell activity. In this way, they reduce the amount of mucosa inflammation but contribute to bacterial persistence and colonization of the genital tract [5, 23].

## 4. Host Immune Response and Susceptibility

Host immune factors are considered the most important determinants of the patients variability in the course and outcome of the disease with the production of pro- or anti-inflammatory cytokines.

Indeed, not only the characteristics of the pathogen, but also host genetics play an important role in determining susceptibility to infections and severity of illness.

Immunogenetic studies in innate immunity can explain individual differences in response to infection [31]. In fact, a low immune response may result in a suitable environment for pathogen colonization, while a strong response could lead to excessive inflammation and tissue damage. In such a way, the modulation of the host response to infection results to be of a great importance for the disease course. TLR agonists or antagonists are under clinical studies for the treatment of cancer and allergies and for the preparation of vaccines and vaccine adjuvants [32].

As far as the outcome of the disease is concerned, genetic studies evaluate the role of genetic variations in immunologically important host genes that can explain individual differences in response to infection. Single nucleotide polymorphisms (SNPs), in which one nucleotide has been substituted or deleted or inserted, are included in the field of immunogenetic research. The interpatient variability is correlated with infections, and the polymorphisms may have important biological consequences either directly or indirectly. The importance of genetic variations in genes encoding TLRs and NODs as potential risk factors for Ct disease depends on the fact that they are involved in the first step of infection by recognizing adequately the specific pathogen among others. TLR2, TLR4, and TLR9 being expressed in the genital human female tract have an important role in the recognition of Ct-PAMPs in infected women. When genetic variations occur in TLR genes, aberrant or dysfunctional receptors may result in an inadequate recognition of Ct and in an increased risk of persistence. Therefore, genetic polymorphisms, which are different and variable among people, can modulate the progress of the disease and determine the susceptibility to Ct genital tract infections. It has been reported that TLR genetic variation may act in both a damaging way (as it is generally supported) and a protective way [33].

NOD genetic variations may be also risk factors for the inadequate recognition and persistence of Ct and, furthermore, could increase the possibility of an aberrant immune response. 

### 4.1. Production of Chemokines and Host Factors

An association between specific host immune response and susceptibility to chlamydial infection has been documented [5].

IFN-*γ* secretion by mucosal cells is found to be significantly higher after stimulation with both Ct-hsp60 and Ct-hsp10 and is correlated with protection against incident *C. trachomatis *infection [34]. CD4 cells result in an increase in cervical mucosa of fertile women whereas CD8 cells are found to be only slightly increased.

Low peripheral blood mononuclear cells (PBMCs) IFN-*γ* and high IL-10 responses to Ct-hsp60 are markers for increased risk of chlamydial infection and PID [7]. In seropositive women, a marked decrease of CD4 T cells leads to a strong risk of PID [35].

Neutrophils and neutrophil defensin levels in cervical secretions correlate with endometritis in women with clinical PID [36]. Reduced IL-2 concentrations in endocervical secretions are strictly related to tubal infertility as well as IL-10 promoter polymorphism (IL-10-1082AA) [37]. Significantly high levels of IL-1 *β*, IL-6, IL-8, and IL-10 are observed in seropositive patients with fertility disorders [11]. IFN-*γ* and IL-12 are positively correlated with fertility in Ct-seropositive patients [1] (Table 2).

## 5. Ct-hsp60

*Chlamydia *heat shock protein 60 (Ct-hsp60) has been investigated as a potential antigen responsible for induction of delayed type hypersensitivity-induced disease. Ct-hsp60 reveals a protective role for vaccination in guinea pig [38]. Detection of elevated titers of antibody to Ct-hsp60 has been found in the subjects with severe disease and mainly in chronic or repeated infections [8]. Ct-hsp60 alone can activate endothelial cells and macrophages to produce adhesion factors and proinflammatory cytokines and to stimulate the secretion of TNF-*α*. TLRs bind to Ct-hsp60 and induce signals for the production of cytokines and chemokines that begin the inflammatory chronic response [4]. Ct-hsp60 is synthesized during infection and is released in the bloodstream. As a consequence, immune cells will produce anti-Ct-hsp60 antibodies.

Hsp60 is an ubiquitous molecule with multiple roles: it is a highly conserved protein and shares numerous identical amino acids between eukaryotes and prokaryotes. This implies that there are common antigenic sites (epitopes) between humans and *Chlamydiae *able to elicit, in infected individuals, cross-reactive antibodies which react, other than with microbial products, also with analogous human antigens (autoimmunity). This pathology is detected in autoimmune diseases such as arthritis, multiple sclerosis, diabetes, atherosclerosis, vasculitis, and thyroiditis [39–41]. Hsp60 is a group I chaperonin, generally mitochondrial molecule that is often associated with its cochaperonin hsp10: hsp60 and hsp10 form a complex of a double ring shape [9]. Upon cell stress and during carcinogenesis, the chaperones are found in the cell surface (sf-hsp60) and/or are secreted from cells into the extracellular space and circulation. The sf-hsp60 can stimulate the autoimmune aggression toward stressed cells and induce the disease development [9]. During persistent infections, Ct produces a large quantity of hsp60 implied in autoimmune disorders [42]. The cross-reactive effects are perpetuated and possibly amplified in human infections due to the fact that human chaperonins are present not only in the cells but also outside them attached to the cell membrane or in circulation. In this case, the risk of autoimmune diseases is increased [43]. Elevated titers of antibodies anti-Ct-hsp60 are recognized as a marker of persistent infection. In fact, a strict correlation between the detection of Ab-Ct-hsp60 and previous chlamydial infection as well as between Ab-Ct-hsp60 and elevated serum Ig G or Ig A levels has been demonstrated [8].

The pathological lesions due to the presence of immunoprecipitates (immunocomplexes formed by anti-Ct-hsp60 antibodies and Ct-hsp60) may furtherly worsen the course of disease forming deposits in several anatomic allocations, such as glomerular basal membrane. 

**Figure 3:**
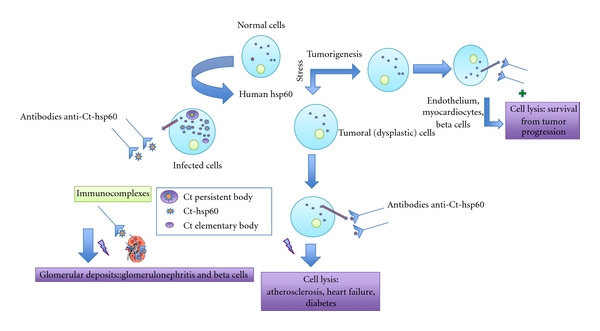
Modified from Cappello et al. 2009 [9]. Potential effects of anti-Ct-hsp60 antibodies. These antibodies recognize surface-hsp60 onstressed or tumor cells, and consequently, they can lead to either damage and persistence of infection or cell lysis producing a regression of certaintypes of cancer. Immunocomplexes (Ct-hsp60 and anti-Ct-hsp60) can cause disease if they form deposits in the renal glomerulus.

Hsp60 may also be elevated in tumors and cardiovascular diseases [44, 45]: in fact superficial (sf) hsp60 occurs in the cell membrane of some tumors being a target for antibodies. If hsp60 is present on the surface of malignant cells, as it happens in some types of cancer, cells with this antigen can be destroyed by anti-hsp60 antibody then leading to damage of the tumor. Following these affirmations, anti-Ct-hsp60 antibodies are prone either to cause a long lasting infection or to be protective against certain tumors stopping the cancer progression [9] (Figure 3).

## 6. Conclusions

Ct immunopathogenesis is a challenge, because the complex mechanism that is on the basis of the interaction between host and microorganism is not fully understood: in fact, host immune response and microorganism virulence strongly affect the disease outcome.

The host immune response does not eliminate the pathogen but in contrast is able to worsen the clinical course of the infection [3–6, 23]; on the other hand, host gene polymorphisms with their anti- or proinflammatory effects address the outcome of *Chlamydia trachomatis *disease. Genetic variation in TLR and NOD genes may affect receptor function, leading to an inadequate recognition of Ct, to an unsuitable immune response and consequently leading to an increased risk of persistence and late sequelae [3]. The determination of genetic polymorphisms may serve to identify persons at high risk of illness development and in need of increased levels of screening and treatment.

The infected nonimmune host cells (epithelial cells) are the first line of human defense. They are involved in the inflammatory response to *Chlamydia*. In fact, they not only induce the influx of inflammatory cells, but also release tissue-damaging molecules. Ct infection stimulates the production of several cytokines such as IL-2, IL-6, IL-8, IL-10, IL1b, TNF-a, GM-CSF, and IFN-*γ* which account for chronic and intense inflammation and for the promotion of cellular proliferation, tissue damage, and tissue remodelling and scarring [4]. CD4 Th1-IFN-*γ* producing cells are strongly involved in the protection from Ct infection and disease, whereas the role of CD8 Th1-cell population in the fertility disorders is not well defined. Hsp60 is an ubiquitous, multifaceted, versatile molecule, which plays a crucial role in Ct pathogenesis. During persistent infections, the pathogen produces a large quantity of hsp60, implied in the pathogenesis of the genital tract and in the autoimmune disorders. The physician should always consider the various roles of hsp60 and anti-hsp60 antibodies and measure them in all cases with suspected or demonstrated autoimmune manifestations. In any case, conclusions about the role of anti-hsp60 antibodies in the onset and progression of disease must be taken as provisory and with need of further investigations. In fact, if, on one side, the anti-Ct-hsp60 are damaging and are likely to cause long-lasting disease, on the other, they can protect from some kinds of cancer.

Further problems that should be deeply examined in Ct infections concern the possible link that may exist between more virulent strains and antibiotic resistance that is known to be a crucial drawback in this disease treatment.

## Figures and Tables

**Table 1:** Presence of Toll-like receptors (TLRs) and nucleotide-binding oligomerization domains (NODs) in the genital tract.

PRRs	PAMPs	Presence in genital tract	
		* In vivo*	* In vitro*
TLR2	Peptidoglycan	+	+
TLR4	LPS and hsp	+	+
TLR9	Bacterial DNA	NT	+
NOD1	Peptidoglycan	NT	NT
NOD2	Peptidoglycan LPS	NT	NT

Modified from den Hartog et al. 2006 [3].

Legenda: hsp: heat shock protein; LPS: lipopolysaccharide; NT: not tested; PAMPs: pathogen-associated molecular patterns; PRRs: pattern recognition receptors.

**Table 2:** Host factors and cellular immune responses associated with susceptibility or protection from infection and/or diseases.

Host factor or response	Association	References
IFN-*γ* production by PBMCs stimulated with Ct-hsp60	Protection from incident *C. trachomatis* infection	[34]
Low PBMC IFN-*γ* and high IL-10 responses to Ct-hsp60	Increased risk of *C. trachomatis* infection and PID	[7]
Low CD4 cell count in HIV-infected women	Increased risk of PID	[35]
Neutrophils and neutrophil defensin levels in cervical secretions	Positive correlation with histologic endometritis in girls with clinical PID	[36]
IL-10 promoter polymorphism (IL-10-1082AA); IL-2 reduction	Increased risk of infertility	[37]
*Cervical cell* production of IL-1*β*, IL-6, IL-8, and IL-10 in response to stimulation with *Chlamydia trachomatis* EBs	Positive correlation with infertility in *C. trachomatis*-seropositive patients	[11]
*Cervical cell* production of IFN-*γ* and IL-12 in response to stimulation with *Chlamydia trachomatis* EBs	Positive correlation with fertility in *C. trachomatis*-seropositive patients	[1]

Legenda: Ct-hsp60: *Chlamydia trachomatis* heat-shock protein 60; EBs: elementary bodies; IFN: interferon; PID: pelvic inflammatory disease; PBMCs: peripheral blood mononuclear cells.
